# The Role of m6A RNA Methylation in Cancer: Implication for Nature Products Anti-Cancer Research

**DOI:** 10.3389/fphar.2022.933332

**Published:** 2022-06-16

**Authors:** Na Song, Kai Cui, Ke Zhang, Jie Yang, Jia Liu, Zhuang Miao, Feiyue Zhao, Hongjing Meng, Lu Chen, Chong Chen, Yushan Li, Minglong Shao, Jinghang Zhang, Haijun Wang

**Affiliations:** ^1^ Department of Pathology, Key Laboratory of Clinical Molecular Pathology, The First Affiliated Hospital of Xinxiang Medical University, Xinxiang, China; ^2^ Department of Pathology, Xinxiang Medical University, Xinxiang, China; ^3^ School of Public Health, Xinxiang Medical University, Xinxiang, China; ^4^ The Second Affiliated Hospital of Xinxiang Medical University, Xinxiang, China

**Keywords:** m6A, RNA methylation, molecular function, signaling pathway, implication, nature products, anti-cancer

## Abstract

N6-methyladenosine (m6A) RNA methylation is identified as the most common, abundant and reversible RNA epigenetic modification in messenger RNA (mRNA) and non-coding RNA, especially within eukaryotic messenger RNAs (mRNAs), which post-transcriptionally directs many important processes of RNA. It has also been demonstrated that m6A modification plays a pivotal role in the occurrence and development of tumors by regulating RNA splicing, localization, translation, stabilization and decay. Growing number of studies have indicated that natural products have outstanding anti-cancer effects of their unique advantages of high efficiency and minimal side effects. However, at present, there are very few research articles to study and explore the relationship between natural products and m6A RNA modification in tumorigenesis. m6A is dynamically deposited, removed, and recognized by m6A methyltransferases (METTL3/14, METTL16, WTAP, RBM15/15B, VIRMA, CBLL1, and ZC3H13, called as “writers”), demethylases (FTO and ALKBH5, called as “erasers”), and m6A-specific binding proteins (YTHDF1/2/3, YTHDC1/2, IGH2BP1/2/3, hnRNPs, eIF3, and FMR1, called as “readers”), respectively. In this review, we summarize the biological function of m6A modification, the role of m6A and the related signaling pathway in cancer, such as AKT, NF-kB, MAPK, ERK, Wnt/β-catenin, STAT, p53, Notch signaling pathway, and so on. Furthermore, we reviewed the current research on nature products in anti-tumor, and further to get a better understanding of the anti-tumor mechanism, thus provide an implication for nature products with anti-cancer research by regulating m6A modification in the future.

## Introduction

In recent years, N^6^-methyladenosine (m6A) RNA modification, as a new regulatory mechanism for controlling gene expression in eukaryotes, has attracted more and more attention and gradually become a hot topic in biological research. Reversible epigenetic modification of m6A RNA was found not only in messenger RNA (mRNA) but also in non-coding RNA (ncRNA). m6A modification affects the biological fate of target RNA molecules and plays an important role in almost biological activities, including the occurrence, development and outcome of cancer (Chen et al., 2019a; He et al., 2019; Sun et al., 2019; Wang et al., 2020e; Huang et al., 2020). Among the more than 100 types of post-transcriptional epitranscriptomic modifications identified in RNAs thus far, m6A is the most abundant internal modification of eukaryotic RNAs, including mRNA, tRNA, rRNA, snRNA, microRNA (miRNA), and long non-coding RNA (lncRNA), contributing to RNA splicing, localization, translation, stabilization, and decay (Gokhale et al., 2016; Wang et al., 2018; Koh et al., 2019; Meyer, 2019; Zhang et al., 2019; Gong et al., 2020). In recent years, it has been found that the abnormal modification of m6A RNA is closely related to the occurrence and development of a variety of tumors (Chen et al., 2018; Jin et al., 2019; Shi et al., 2020; Wang et al., 2020a; Zhang et al., 2021a). Therefore, it is necessary to conduct in-depth studies on the relationship between m6A and tumor, especially to harness the key mechanisms involved in m6A modification for anti-cancer research (Niu et al., 2018; Gao et al., 2021).

Natural products are essential to modern medicine, such as curcumin, chrysin, cucurbitacin B, cinnamaldehyde, bruceine D, baicalein, epigallocatechin gallate, fucoidan, resveratrol, rhein, and so on. Many of which have been shown to effectively regulate the progression of cancer and enhance the anticancer effects of various anticancer drugs (Li et al., 2020a; Chen et al., 2020b; Chen et al., 2021b; Zhang et al., 2021b; Wang et al., 2021). In addition, 73 percent of antibiotics, 49 percent of anticancer compounds, and 32 percent of all new drugs approved by the U.S. Food and Drug Administration (FDA) between 1980 and 2012 were natural products or derivatives of them (Newman and Cragg, 2016; Harvey et al., 2018). These natural products, including compounds with antibiotic, antifungal, immunosuppressant, and anticancer activity, are a rich source of anticancer agents and provide new and more effective anticancer agents for therapeutic use. However, there are few reports that natural products inhibit tumor genesis and development by regulating m6A RNA modification (Qian et al., 2021b; Feng et al., 2022; Jiang et al., 2022).

In this review, we summarize the biological functions of m6A modification, the role of m6A in tumors, and the related signal pathways of m6A in tumors, such as Akt, NF-kB, MAPK, ERK, and Wnt/β-Catenin, STAT, p53, Notch signaling pathway, etc. Moreover, we also review the research progress of natural products in antitumor, and further understands its antitumor mechanism, so as to provide an implication for the anti-cancer research of natural products *via* regulating m6A modification in the future.

## Part 1: Overview of m6A RNA Methylation

### m6A RNA Methyltransferases—Writers

The RNA m6A modification is deposited by the m6A methyltransferase complex (called as “writer”), which mainly consist of a methyltransferases like 3 and 14 (METTL3 and METTL14) heterodimeric enzymatic core, and other cofactor protein, such as Wilm’s tumor-1-associated protein (WTAP), RNA binding motif protein 15 (RBM15) and its paralogue RBM15B, vir like m6A methyltransferase associated (VIRMA, originally known as KIAA1429, Virilizer), zinc finger CCCH type containing 13 (ZC3H13), and CBLL1 (Patil et al., 2016; Roundtree et al., 2017a; Table 1).

**TABLE 1:** Functions of m6A regulators in RNA metabolism.

Type	Regulators	Biology function
m6A writer	METLL3	Catalyzes m6A modification
	METLL14	Cooperates METLL3 to recognize the subtract
	METLL16	Catalyzes m6A modification
	WTAP	Promotes METLL3/14 heterodimer to the nuclear speckle
	VIRMA	Recruits the m6A complex to the special RNA site and interacts with polyadenylation cleavage factors CPSF5 and CPSF6
	ZC3H13	Promotes the nuclear localization of the m6A complex
	RBM 15	Binds RNA and recruits m6A complex to special RNA site
	CBLL1/Hakai	Core component for METTL3/14 stabilization
m6A eraser	ALKBH5	Removes m6A modification
	FTO	Removes m6A modification
m6A reader	YTHDC1	Contributes to RNA splicing, stabilization, export
	YTHDC2	Enhances mRNA translation and reduces the abundance of target RNA
	YTHDF1	Enhances mRNA translation
	YTHDF2	Promotes mRNA degradation
	YTHDF3	Cooperates YTHDF1/2 for RNA translation/degradation
	IGF2BPs	Enhances mRNA stability and storage
	elF3	Enhances mRNA translation
	hnRNPA2B1	Mediates pri-miRNA processing and pre-mRNA splicing
	hnRNPC	Mediates pre-mRNA splicing

METTL3 and METTL14 are essential components of methyltransferase complex (MTC), in which METTL3 is catalytically active while METTL14 has critical structural scaffold functions, isolated METTL3 without METTL14 is not bioactive (Roundtree et al., 2017a; Hsu et al., 2017). WTAP, as the key METTL3 adaptor, directly interacts with METTL3 and recruits METTL3-METTL14 heterodimeric complex to nuclear speckles for m6A modification (Ping et al., 2014). Meanwhile, WTAP also binds to the m6A consensus RRACH (R = A/G, H = A/C/U) motif of RNA and recruits catalytic subunits METTL3 and METTL14 to the vicinity of the target RNA sequence (Ping et al., 2014). In addition, studies have shown that knockdown of WTAP reduces METTL3 and METTL14, while knockdown of METTL3 or METTL14 does not reduce or even increase WTAP, although a single knockdown of METTL3 or METTL14 reduces each other (Ping et al., 2014; Kobayashi et al., 2018). Moreover, WTAP also may scaffold the MTC and RBM15/RBM15B recruits the MTC to target sites of modification (Kan et al., 2017). METTL16 is another independent m6A methyltransferase that can directly methylate UACAGAGAA motifs. In addition, METTL16 can also regulate the activity of all cellular methyltransferases including METTL3/METTL14, so the deletion of METTLl6 can reduce the methylation level of UACAGAGAA and/or RRACH motif regions (Fazi and Fatica, 2019; Koh et al., 2019; Aoyama et al., 2020). Recent studies have shown that METTL5 as the enzyme responsible for 18s rRNA m6A modification in site of 1831A, which must form a METTL5-TRMT112 (TRMT112, a methyltransferase activator) heterodimeric complex to maintain its biological stability (Van Tran et al., 2019; Rong et al., 2020).

RNA m6A modification is enriched in the 3′untranslated region (3′UTR) and near termination codon of mature polyadenylate mRNA in mammalian system, and plays a regulatory role in the transcription of eukaryotic mRNA. VIRMA, an important WTAP interactor, is essential for the deposition of m6A to the 3′UTR of mRNA and near stop codons and recruiting the methyltransferase core components METTL3/METTL14/WTAP (Horiuchi et al., 2013; Yue et al., 2018; Shi et al., 2019; UniProt, 2019); Knockdown of VIRMA resulted in reduced m6A methylation near the 3′UTR of the stop codon. In addition, VIRMA binds to the polyadenylation cleavage factors CPSF5 and CPSF6 in an RNA-dependent manner, and knockdown of CPSF5 also resulted in a significant shortening of the 3′UTR of the mRNA (Yue et al., 2018).

ZC3H13 could promote nuclear localization of the writer complex for RNA m6A modification, ZCCHC4 as the 28s rRNA modification enzyme for m6A modification at the site of 4220A (Ren et al., 2019). RBM15/15B binds to U-rich regions and cooperates with MTC for certain RNAs methylation (Shi et al., 2019). CBLL1 (Cbl proto-oncogene like 1), also known as Hakai, was originally found to encode the E3 ubiquitin ligase for the E-cadherin complex and mediate its ubiquitination, endocytosis, and degradation in lysosomes. Recent studies have shown that CBLL1 is required for stabilization of core components METTL3/14 of the m6A RNA methylation and plays a role in the efficiency of mRNA splicing and RNA processing (Yue et al., 2018; Bawankar et al., 2021).

### m6A RNA Demethylases—Erasers

The intracellular RNA m6A demethylation is mainly accomplished by fat mass and obesity-associated protein (FTO) and the alkylation repair homolog protein 5 (ALKBH5), referred to as erasers, which cooperates with m6A methylases to maintain the dynamic balance of m6A modification in the cells (Jia et al., 2011; Zheng et al., 2013; Xue et al., 2019).

As the first m6A mRNA demethylase, FTO established the concept of reversible RNA modification (Jiang et al., 2021b). The whole transcriptome RNA demethylation analysis clarified that FTO is a potent nuclear mRNA processing regulator, which preferentially binds to the pre-mRNA in the intron region, in the proximity of alternative splicing exons and poly(A) sites, and participates in alternative splicing and 3′ end mRNA processing (Bartosovic et al., 2017). In addition, multiple evidences indicate that FTO does play a key role in regulating fat mass, fat production and body weight (Merritt et al., 2018). Epidemiological studies also show that FTO SNPs is strongly associated with increased risk of various cancers (Chen and Du, 2019). FTO has recently been shown to play an m6A-dependent role in multiple biological processes, such as cancer cell apoptosis, proliferation, migration, invasion, metastasis, cell-cycle, differentiation, stem cell self-renewal and so on (Deng et al., 2018; Wang et al., 2020d). In addition, studies have shown that FTO binds multiple RNA species, including mRNA, snRNA, and tRNA, and can demethylate internal m6A and cap m6Am in mRNA, internal and cap m6Am in snRNA, internal m6A in U6 RNA, and N1-methyladenosine (m1A) in tRNA (Wei et al., 2018).

ALKBH5 is another major m6A demethylase which plays a critical biological and pharmacological role in human cancer. ALKBH5 is an independent prognostic indicator in a variety of cancers. ALKBH5 can regulate various tumor biological processes, such as the cell proliferation, invasion, migration, metastasis, cancer stem cell self-renewal and tumor microenvironment (Li et al., 2020b; Shen et al., 2020). In addition, ALKBH5 also plays an important role in human non-cancer diseases, such as reproductive system diseases (Nettersheim et al., 2019). The potential regulatory mechanism of ALKBH5 relies on m6A-dependent modifications (Wang et al., 2020c). Moreover, it was found that ALKBH5 knockout resulted in increased mRNA m6A modification level in the nucleus, but a decrease in the amount of mRNA in the cytoplasm, suggesting that ALKBH5 may be involved in mRNA transport (Bartosovic et al., 2017). In addition, neither FTO nor ALKBH5 display a preference for RRACH m6A motif demethylation (Zou et al., 2016).

### m6A RNA Binding Proteins—Readers

The m6A modification plays an important role in the production, processing, splicing and translation of mRNA (Huang et al., 2021a). m6A binding protein (also called: reader) is essential for the recognition of m6A RNA, including m6A direct binding proteins (such as: YTH domain family, IGF2BPs, eIF3, etc) and m6A indirect binding proteins (hnRNP family, etc).

At present, it is considered that the YT521-B homology (YTH) domain family consists of YTH domain containing protein 1-2 (YTHDC1-2) and YTH domain family protein 1-3 (YTHDF1-3). YTHDC1 mediates the splicing of m6A pre-mRNA *via* recruiting splicing factor and nuclear export adaptor SRSF3 while blocking SRSF10 (Xiao et al., 2016), and NXF1 also involved the nuclear exporting of m6A-containing mRNAs (Roundtree et al., 2017b); ELAVL1 (ELAV like RNA binding protein 1, also known as HuR) is known to be an RNA stabilizing protein that interacts with mRNAs containing m6A modifications and cooperates with YTHDC1 to enhance the stability and subsequent translation of target gene mRNAs (Liang et al., 2021). The m6A reader YTHDC2 functions in the cytoplasm, affecting the m6A mRNA translation efficiency and abundance of its targets. It has been proposed that YTHDC2 interacts with m6A-containing mRNAs, ribosomes, and XRN1 to regulate mRNA stability and translation. In addition, MEIOC also can be bound to YTHDC2 in an RNA-independent manner, stabilize the binding of YTHDC2 to mRNA (Abby et al., 2016; Inada, 2020). YTHDF3 has been shown to promote the functions of YTHDF1 and YTHDF2, which promote m6A mRNA translation by binding to YTHDF1 and enhance RNA degradation by interacting with YTHDF2, respectively (Shi et al., 2017).

The insulin-like growth factor-2 (IGF2) mRNA-binding proteins 1, 2, and 3 (IGF2BP1/2/3) as a distinct family of m6A readers that regulate mRNA stability, transport, and translation (Uyar et al., 2017). Recent research shows that the association between IGF2BPs and target mRNAs, such as myc mRNA, is enhanced by m6A modification of target transcripts, indicating that IGF2BPs is an important m6A reader (Muller et al., 2019). In addition, IGF2BPs can stabilize the mRNA of target genes by recruiting and interacting with RNA stabilizing proteins, such as: ELAVL1/HuR, MATK3, PABPC1, etc (He et al., 2019; Zhu et al., 2020a), and also IGF2BPs can protect the target mRNA from stress condition (Huang et al., 2018). Eukaryotic initiation factor 3 (eIF3) can be regarded as a reader of 5′ UTR m6A, which can interact with m6A modified RNA, enhance ribosomal loading and promote the translation of target gene mRNA (Shah et al., 2017).

**FIGURE 1:**
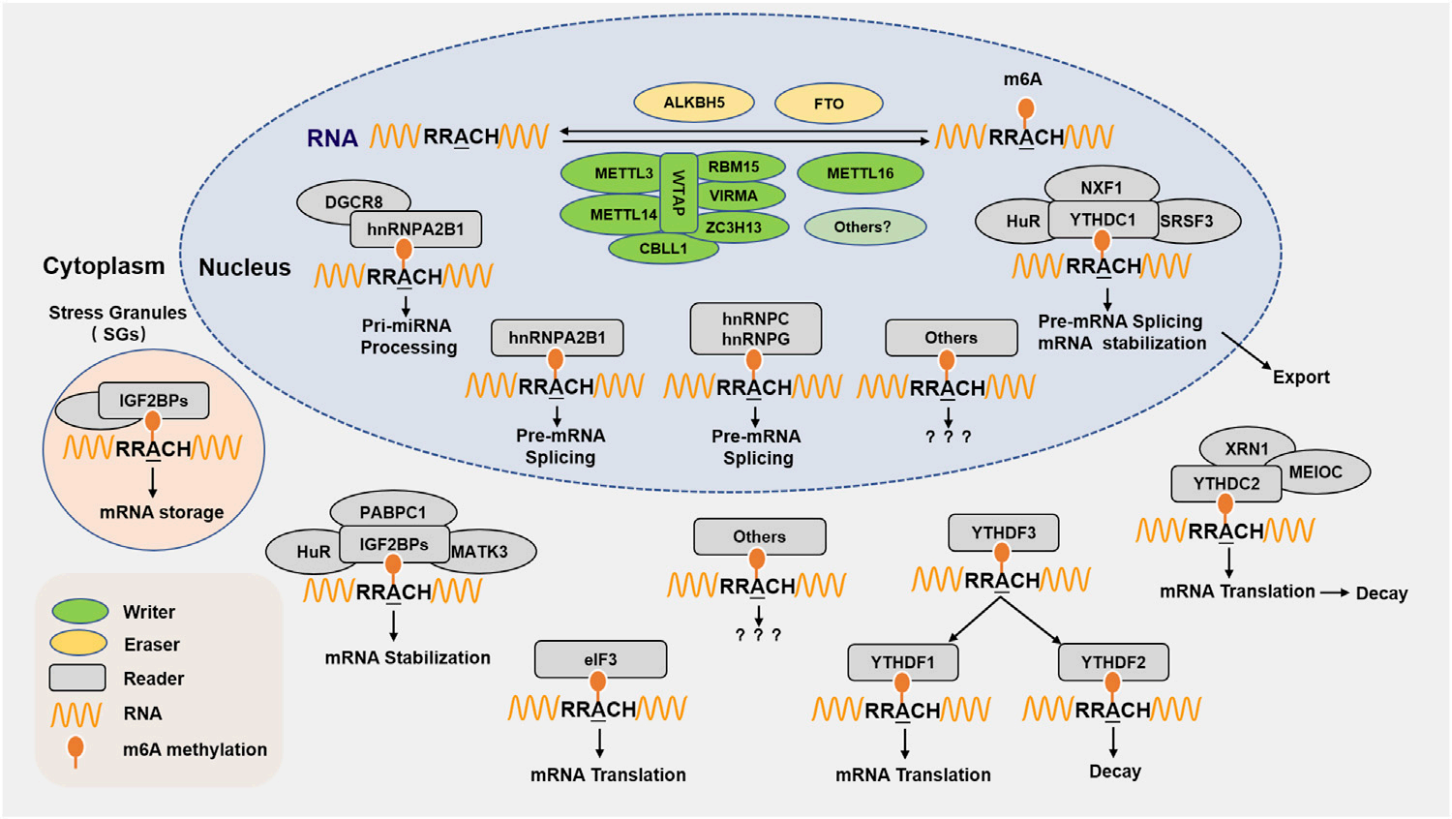
m6A RNA methylation regulating proteins and molecular functions. m6A RNA methylation is performed by its writer, eraser, and reader to add, delete, recognize m6A, respectively. The writer refers to m6A methyltransferases including METTL3, METTL14, WTAP, RBM15/15B, VIRMA/KIAA1429/Virilizer, ZC3H13, CBLL1/Hakai, and METTL16. The m6A eraser is the demethylase FTO and ALKBH5. The m6A readers are proteins that recognize m6A sites and perform a variety of functions in the nucleus or cytoplasm, the currently identified m6A reader proteins including YTHDC1, YTHDC2, YTHDF1, YTHDF2, YTHDF3, IGF2BPs, eIF3, and hnRNPA2B1, HNRNPC, HNRNPG. m6A methylation is involved multiple functions in RNA metabolism, including RNA stabilization and splicing, miRNA processing, nuclear export, translation, RNA storage and decay, and so on.

Heterogeneous ribonucleoproteins (hnRNPs) refer to a large family of RNA-binding proteins (RBPs) that are involved in multiple aspects of nucleic acid metabolism, including alternative splicing, processing, mRNA stabilization, and subsequent transcription and translation. Among them, hnRNPA2B1 can directly bind to m6A RNA to regulate RNA splicing and processing. In addition, hnRNPA2B1 can also cooperate with proteins, such as: DGCR8, etc., to promote the processing and formation of miRNA (Alarcon et al., 2015). The m6A reader protein hnRNPC or hnRNPG binds m6A RNAs through an “m6A switch” mechanism, in which m6A-mediated RNA hairpin destabilization exposes a single-stranded hnRNPC or hnRNPG-binding motif, and binds to m6A RNA and facilitates processing (Liu et al., 2017; Figure 1).

## Part 2: The Role of m6A Modification in Cancer

More and more research evidences show that m6A modification is related to the proliferation, differentiation, tumorigenesis, invasion and metastasis of tumors, and plays a key role as an oncogene or tumor suppressor gene in the occurrence and development of malignant tumors (Table 2).

**TABLE 2:** Role of m6A modification in various cancers.

Cancer	Cell lines	Expression	Function	Role	Target/Pathway	Mechanism	References
AML	MOLM13	METTL3↑	Writer	Oncogene	c-Myc↑, BCL2↑, PTEN↑	Inhibit AML cell differentiation and apoptosis	Vu et al. (2017)
	K562, Kasumi-1	METTL3/14↑	Writer	Oncogene	MDM2↑, P21↓, P53↓	Promote cell proliferation and cell cycle, inhibit AML cell apoptosis and differentiation	Sang et al. (2022)
	AML-MSCs	METTL3↓	Writer	Suppressor	AKT↑, PI3K↑-AKT↑	Upregulate PI3K-AKT pathway activity and enhance chemoresistance	Pan et al. (2021b)
	MMC6, NOMO1	ALKBH5↑	Eraser	Oncogene	TACC3↑	Maintenance of AML and self-renewal of LSCs/LICs	Shen et al. (2020)
	MOLM13, THP1	ALKBH5↑	Eraser	Oncogene	AXL↑, p-SRC↑, p-AKT↑, p-ERK1/2↑, p-STAT3↑	Affects mRNA stability of AXL; Maintenance of AML and self-renewal of LSCs/LICs	Wang et al. (2020b)
	MonoMac-6, MV4-11, NB4	FTO↑	Eraser	Oncogene	ASB↓, RARA↓	Enhances cell transformation, leukemogenesis, and inhibits differentiation	Li et al. (2017)
	NB-4, MOLM13	YTHDC1↑	Reader	Oncogene	MCM4↑	Maintenance of AML and self-renewal of LSCs, YTHDC1 upregulates MCM4 expression and promotes AML	Sheng et al. (2021)
	MOLM13	YTHDC1↑	Reader	Oncogene	Myc↑, Phase seperation↑	Promotes liquid-liquid phase separation, maintains AML cell survival and the undifferentiated state	Cheng et al. (2021)
NSCLC	A549, A520; H1299, H1975	METTL3↑	Writer	Oncogene	c-Myc↑, BCL2↑	Promotes cell growth, invasion and migration	Wu et al. (2021a); Zhang et al. (2021a)
	A549	METTL3↑	Writer	Oncogene	EGFR↑, TAZ↑, YAP↑, MAPKAPK2↑, DNMT3A↑	Promotes cell growth, survival, invasion and migration	Lin et al. (2016); Jin et al. (2019)
	A549, LC-2/ad	METTL3↑	Writer	Oncogene	JUNB↑, E-Cadherin↓, FN1↑, VIM↑	Promotes EMT by up-regulating JUNB expression, which upregulates EMT-related protein expression	Wanna-Udom et al. (2020)
	A549, Calu1	METTL3↑	Writer	Oncogene	LCAT3↑-FUBP1↑-MYC↑	Enhances LCAT3 lncRNA stabilization Promotes cell proliferation, migration and invasion	Qian et al. (2021a)
	A549, H838	METTL3↑	Writer	Oncogene	DAPK2↓, NF-kB↑	Inhibiting DAPK2 expression and activating NF-κB pathway	Jin et al. (2019)
	A549, H1299, H1975	METTL3↑	Writer	Oncogene	miR-1246↑, PEG3↓; miR-143-3p↑, VASH1↓, dY-Tub↓	Promotes cell growth, EMT, invasion, migration and angiogenesis	Huang et al. (2021a) ; Wang et al. (2020b)
	A549, H1975	METTL3↑	Writer	Oncogene	miR-1915-3p↓, SET↑, JNK/Jun & NF-κB↑	Promotes cell growth, EMT, survival, invasion, and migration	Pan et al. (2021a)
	H1975, H322	METTL3↑	Writer	Oncogene	SLC7A11↑	Enhances the stability of SLC7A11 mRNA which promotes cell proliferation and inhibits ferroptosis	Xu et al. (2022)
	H1299, H1650	METTL3↑	Writer	Oncogene	LncRNA ABH11-AS1↑, EZH2-KLF4, Warburg effect↑	Enhance the Warburg effect and promote tumor progression	Xue et al. (2021)
	H1975, H1299	YTHDC2↓	Reader	Suppressor	SLC7A11↑	Downregulation of YTHDC2 expression leads to enhanced SLC7A11 mRNA stability and promotes tumorigenesis	Ma et al. (2021a)
	H441, H1299	YTHDC2↓	Reader	Suppressor	HOXA13↑, SLC3A2↑	Downregulation of YTHDC2 expression leads to enhanced HOXA13 mRNA stability and promotes tumorigenesis	Ma et al. (2021b)
	HCC827	METTL3↓	Writer	Suppressor	Bax↓, cleaved caspase 3↓, FBXW7↓, c-Myc↑, Mcl-1↑	Downregulation of METTL3 decreased apoptotic molecules and increased FBXW7 expression and its target genes Mcl-1 and c-Myc, and promotes tumorigenesis	Wu et al. (2021b)
	A549, H1299	YTHDF1,3↑	Reader	Oncogene	YAP↑, ABCG2↑, ERCC1↑, CTGF↑, Cyr61↑	Promotes cell growth, EMT, invasion, migration and anti-apoptosis	Jin et al. (2019)
HCC	HepG2, Huh7	METTL3↑	Writer	Oncogene	SOCS2↓, JAK/STAT↑	Promotes cell growth, migration, metastasis, tumorgenicity	Chen et al. (2018)
	Huh7, HCCLM3	WTAP↑	Writer	Oncogene	ETS1↓, ETS1-p21/p27↓	Promotes cell growth, tumorgenicity	Chen et al. (2019a)
	SK-Hep1, HCCLM3	VIRMA↑	Writer	Oncogene	GATA3↓	Enhances tumor growth and metastasis	Lan et al. (2019)
	HepG2, Huh7	METTL14↓	Writer	Suppressor	USP48↓, SIRT6↓, aerobic glycolysis↑	Down-regulation of METTL4 in HCC promotes aerobic glycolysis, cell proliferation, tumorigenesis	Du et al. (2021)
	HepG2	METTL14↓	Writer	Suppressor	miR-126↓	Downregulated METTL14 affects miR-126 processing and promotes tumor cell metastasis	Ma et al. (2017)
	Huh7, MHCC97H	ALKBH5↓	Eraser	Suppressor	LYPD1↑	Downregulated ALKBH5 enhances LYPD1 mRNA stability and translation, which promotes cell growth, invasion	Chen et al. (2020a)
	MHCC97H, HCCLM3	FTO↑	Eraser	Oncogene	SOX2↑, KLF4↑, NANOG↑	Promotes cell growth, metastasis, stemness	Bian et al. (2021)
	Huh7, MHCC97H	YTHDF1↑	Reader	Oncogene	PI3K-AKT-mTOR↑	Promotes cell proliferation, EMT, migration, invasion, and cell cycle process	Luo et al. (2021)
	HepG2, Huh7	IGF2BP2↑	Reader	Oncogene	FEN1↑	Enhances FEN1 mRNA stability and promotes cell proliferation	Pu et al. (2020)
Breast cancer	MDA-MB-231, MCF7, BT549	METTL3↑	Writer	Oncogene	BCL2↑; SOX2↑; KRT7↑; MALAT1↑, HMGA2↑; PD-L1↑	Promotes cell proliferation, EMT, metastasis, stemness; Anti-apoptosis, inhibits tumor immunity	Wang et al. (2020a) *etc*.
	—	METTL14↓ ZC3H13↓	Writer	Suppressor	APC↓, Wnt signaling↑	Promotes cell proliferation, EMT, migration, invasion, metastasis	Gong et al. (2020)
	MDA-MB-231, MCF7; SKBR3 MDA-MB-453	FTO↑	Eraser	Oncogene	BNIP3↓; miR-181-3P↓-ARL5B↑	Promotes cell proliferation, EMT, migration, invasion, metastasis	Niu et al. (2019); Xu et al. (2020)
	MDA-MB-231, MCF7	YTHDF1↑	Reader	Oncogene	FOXM1↑	Enhances FOXM1 translation, Promotes cell proliferation, invasion, metastasis	Chen et al. (2022)
	MDA-MB-231 4T1	YTHDF3↑	Reader	Oncogene	ST6GALNAC5↑, GJA1↑, EGFR↑, VEGFRA↑	Promotes brain metastasis, invasion, angiogenesis	Chang et al. (2020)

### Acute Myeloid Leukemia

The link between m6A and tumorigenesis was first demonstrated in AML, where overexpression of methyltransferases leads to increased RNA m6A modification, leading to abnormal hematopoietic stem cell differentiation and promoting leukemogenesis (Vu et al., 2017).

The abnormally high expression of METTL3 in AML can promote m6A modification and promote the occurrence and development of AML by enhancing the translation of c-MYC, BCL2, and PTEN mRNAs. Moreover, knockdown of METTL3 can lead to increased p-AKT levels and promote cell differentiation (Vu et al., 2017). In addition, METTL3 and METTL14 exert oncogenic roles in AML by increasing m6A levels in MDM2 mRNA and targeting the MDM2/p53 signaling pathway (Sang et al., 2022). However, the low expression of METTL3 in AML-MSCs (mesenchymal stem cells from AML patients) promotes the expression of AKT protein and upregulate the PI3K-AKT signaling pathway, which ultimately leads to the enhanced adipogenesis ability of AML-MSCs and facilitates the chemoresistance of AML (Pan et al., 2021b).

ALKBH5 is aberrantly overexpressed in AML and associated with poor prognosis. ALKBH5 exerts tumor-promoting roles in the development and maintenance of AML and self-renewal of leukemia stem/initiating cells (LSCs/LICs) through post-transcriptional regulation of its key target TACC3, AXL (Wang et al., 2020b; Shen et al., 2020). FTO is highly expressed in AML containing t (11q23)/MLL rearrangements, t (15;17)/PML-RARA, FLT3-ITD and/or NPM1 mutations. FTO inhibits the expression of ASB2 and RARA by reducing m6A levels of the target mRNA, which in turn enhances leukemic oncogene-mediated cell transformation and leukemogenesis, and inhibits all-trans retinoic acid (ATRA)-induced AML cell differentiation (Li et al., 2017).

YTHDC1 is overexpressed in AML and is required for proliferation, differentiation and development, maintenance of self-renewal, leukemogenesis, and survival (Sheng et al., 2021). Moreover, YTHDC1 can also promote the development of AML by promoting phase separation. In recent years, studies have shown that abnormal phase separation is closely related to the occurrence of tumors. YTHDC1 binds to m6A and forms nuclear condensates (nYACs) which are essential for maintaining targets mRNA stability and AML cell survival and the undifferentiated state (Cheng et al., 2021).

### Non-Small-Cell Lung Cancer

In non-small-cell lung cancer (NSCLC), METTL3 promotes the translation of mRNAs of certain genes by increasing m6A levels, including c-Myc, BCL-2, epidermal growth factor receptor (EGFR), hippo pathway effector TAZ and YAP, MAPKAPK2 (MK2), and DNMT3A, thereby promoting lung adenocarcinoma (LUAD) cell proliferation, survival, invasion and metastasis (Lin et al., 2016; Jin et al., 2019; Wu et al., 2021a; Zhang et al., 2021c). High expression of METTL3 can improve the stability of JUNB mRNA and increase its expression. JUNB is an important transcriptional regulator of epithelial-mesenchymal transition (EMT), including CDH1/E-cadherin, FN1/Fibronectin and VIM/Vimentin (Wanna-Udom et al., 2020). Long non-coding RNAs (lncRNAs) play key roles in a variety of physiological and pathological processes. METTL3 enhances the stability of LCAT3 lncRNA through m6A modification, and then promotes the proliferation, invasion and metastasis of LUAD cells through the LCAT3-FUBP1-MYC axis (Qian et al., 2021a). Moreover, METTL3 also upregulates the level of miR-1246 which reduces the expression of the tumor suppressor PEG3 to promote the development of lung adenocarcinoma (Huang et al., 2021b); In addition, METTL3 can also promote tumor proliferation, EMT, invasion, and migration by down-regulating miRNAs, such as: miR-1915-3p (Pan et al., 2021a). Cell ferroptosis, a novel form of programmed cell death. The highly expressed METTL3 in LUAD can promote LUAD cell proliferation and inhibit ferroptosis, and its molecular mechanism is that METTL3-mediated m6A modification enhances the stability of SLC7A11 mRNA and promotes its translation (Xu et al., 2022). Metabolic reprogramming, known as the Warburg effect, is considered a key hallmark of cancer, including lung cancer. Studies have shown that METTL3 can enhance the stability of the lncRNA ABH11-AS1 transcript, thereby enhancing the Warburg effect of lung adenocarcinoma, and ultimately promoting the occurrence and development of tumors (Xue et al., 2021).

The m6A reader YTHDC2 is frequently repressed in LUAD, indicating a poor prognosis. Downregulation of YTHDC2 expression leads to enhanced SLC7A11 and SLC3A2 mRNA stability and promotes tumorigenesis (Ma et al., 2021a; Ma et al., 2021b). But there is an interesting study showing that METTL3 has an anti-tumor effect, downregulation of METTL3 decreased protein levels of apoptotic molecules and increased protein levels of the FBXW7 gene and its target genes Mcl-1 and c-Myc, and promotes tumorigenesis in LUAD which may be caused by the heterogeneity of tumor cells (Wu et al., 2021b). METTL3, YTHDF1/3 is highly expressed in LUAD, METTL3 promotes YAP mRNA translation by recruiting YTHDF1/3 and eIF3b to the translation start site and increased YAP mRNA stability by modulating the MALAT1-miR-1914-3p-YAP axis, ultimately resulting in increased YAP expression and activity that induces NSCLC drug resistance and metastasis (Jin et al., 2019).

### Hepatocellular Carcinoma

METTL3 is highly expressed in HCC and is closely associated with poor prognosis of HCC patients, and promotes HCC cell proliferation, migration, metastasis and tumorgenicity *via* YTHDF2-dependent post-transcriptional silencing of SOCS2, which is a member of the suppressor of cytokine signaling (SOCS) family, and is a negative regulator of the JAK/STAT pathway (Chen et al., 2018). WTAP-guided m6A modification plays a key role in HCC oncogenesis through the HuR1-ETS1-P21/P27 axis (Chen et al., 2019b). High expression of VIRMA in HCC patients is associated with poor prognosis. VIRMA induces m6A methylation of the 3′ UTR of GATA3 pre-mRNA, leading to dissociation of the RNA-binding protein HuR and degradation of GATA3 pre-mRNA, promoting tumor growth and metastasis *in vivo* (Lan et al., 2019).

However, METTL14, as a tumor suppressor in HCC, is down-regulated. The stability of USP48 mRNA is reduced due to decreased m6A modification of METTL14 and leads to the degradation of its downstream target gene SIRT6, which ultimately enhances aerobic glycolysis in tumor cells (Du et al., 2021). In addition, METTL14, as an anti-metastatic factor, acts as a favorable factor for HCC by regulating m6A-dependent miRNA processing (Ma et al., 2017).

Downregulation of ALKBH5 is associated with poor prognosis in HCC. Decreased expression of ALKBH5 leads to increased 3′UTR m6A modification of LYPD1 mRNA, which in turn results in the increased expression of LYPD1, which promotes tumor cell growth, migration, invasion and metastasis (Chen et al., 2020c). FTO can increase the proportion of stem-like cells in HCC by enhancing the expression of SOX2, KLF4, and NANOG (Bian et al., 2021).

YTHDF1 is highly expressed in HCC and associated with poor survival. YTHDF1 participates in the progression of HCC by activating the PI3K/AKT/mTOR signaling pathway and inducing EMT (Luo et al., 2021). Overexpressed IGF2BP2 can directly recognize and bind to the m6A site of FEN1 mRNA, enhancing the stability of FEN1 mRNA and promoting the proliferation of liver cancer cells *in vitro* and *in vivo* (Pu et al., 2020).

### Breast Cancer

m6A regulatory factor is an important participant in the malignant progression of breast cancer and may be a prognostic and therapeutic target for BCa. METTL3 is highly up-regulated in BCa and predicts a poor prognosis. High expression of METTL3 can increase the modification level of downstream target mRNA 3′UTR m6A and enhance the stability and translation of mRNA, such as BCL-2 (Wang et al., 2020a), SOX2 (Xie et al., 2021), KRT7 (Chen et al., 2021a), MALAT1 (Zhao et al., 2021), etc., which can promote BCa cell proliferation, EMT, invasion, metastasis, tumorigenicity and stem cell. Tumor immune deficiency is an important cause of tumorigenesis. PD-L1, the ligand of PD-1, is highly expressed in many cancers and is closely associated with mortality in cancer patients, establishing the role of PD-1 in cancer-induced immunosuppression. METTL3 enhances the stability of PD-L1 mRNA, promotes the expression of PD-L1 and induces tumor immunosuppression by relying on m6A modification (Wan et al., 2022). However, METTL3 is low expression in triple negative breast cancer (TNBC) and strongly associated with short-distance-metastasis-free survival. METTL3 knockout can enhance cell migration, invasion and adhesion by reducing m6A level (Shi et al., 2020). Downregulation of METTL14 and ZC3H13 in BCa, act as tumor suppressor, has been found in breast cancer and poor prognosis is predicted (Gong et al., 2020).

High levels of FTO were significantly associated with lower survival in BCa patients. FTO promotes BCa cell proliferation, colony formation and metastasis *in vitro* and *in vivo* (Niu et al., 2019; Xu et al., 2020).

YTHDF1 and YTHDF3 are highly expressed in BCa patients and with poor prognosis, which can enhance the expression of related genes, such as FOXM1, ST6GALNAC5, GJA1, EGFR, VEGFRA, etc., and promote the metastasis, invasion and angiogenesis of BCa cells (Chang et al., 2020; Chen et al., 2022).

### The Diverse Role of m6A in Cancer

More recently, extensive efforts have been made to investigate the biological effects of dysregulated m6A modifications and related mechanisms (i.e., m6A writers, erasers, and reader proteins) in various cancers, such as prostate cancer (Wang et al., 2022), colorectal cancer (Tanabe et al., 2016; Zhu et al., 2020b), gastric cancer (Xu et al., 2021), esophageal cancer (Guo et al., 2020), cervical cancer and endometrial cancer (Zhang and Yang, 2021; Huang et al., 2022), pancreatic cancer (Li et al., 2021), skin cancer (Yang et al., 2021), etc. Moreover, METTL16, as an independent regulator, plays an important role in RNA m6A modification. In the nucleus, METTL16 functions as a writer for the deposition of m6A in its specific mRNA; And in the in the cytoplasm, METTL16 could promote the translation of over 4000 mRNA transcripts *via* interacting with eIF3a and 3b (Su et al., 2022). In addition, m6A regulator also involved the immune response. There is evidence that the m6A eraser FTO can decrease the response of PD-1 inhibitor immunotherapy and promote tumorigenesis. Knockdown of FTO enhances the sensitivity to interferon gamma (IFNγ) and anti-PD-1 therapy (Yang et al., 2019). Furthermore, FTO could promote the malignant transformation and tumorigenesis *via* up-regulated the target gene NEDD4L in keratinocytes. Upon further investigation, it was found that the FTO expression was elevated after treated with low-level arsenic which is a human carcinogen (Cui et al., 2021).

Above all, different m6A regulators have different biological effects in various tumors. Even if the same regulator is in the same tumor but is of different molecular subtypes, the biological effects are also not the same, which may be related to tumor heterogeneity and tumor microenvironment. In general, m6A plays an important role in the regulation of tumor cell proliferation, cell cycle, EMT, invasion, metastasis, stemness maintenance, apoptosis, immune effect, and other aspects, which is of great significance for the occurrence and development of tumors.

## Part 3: The Anti-Cancer Effect of Nature Products by Regulating m6A Modification

Natural products and compounds derived from natural products account for about 40% of all drugs approved for clinical use, among which ∼70% of anticancer drugs are derived from natural products. (Butler et al., 2014; Newman and Cragg, 2016; Bahman et al., 2018; Sahm et al., 2020). After years of efforts, natural products have also made great progress in anti-cancer, and many natural small molecules have been successfully marketed and become star therapeutic drugs in the corresponding field. For example, paclitaxel, camptothecin, homoharringtonine, ginsenoside Rg3, etc. In recent years, with the deepening of understanding and attention to natural products, many new opportunities have been brought to the development of natural anti-tumor molecules (Singla et al., 2021a; Singla et al., 2021b; Tan et al., 2021; Wang et al., 2021; Singla et al., 2022).

### β-Elemene

Elemene, a sesquiterpenoid natural compound, is the extraction of traditional Chinese medicinal material herb Curcuma wenyujin, and which is a mixture of β-, γ-, δ-elemene with β-elemene as the main component. β-elemene, the most important component of elemene for pharmaceutical activity, has been shown to be effective *in vitro* and *in vivo* against a variety of cancers, such as lung, leukemia, liver cancer, gastric, cervical cancer, colorectal, ovarian, glioblastoma, and melanoma, and so on (Li et al., 2013; Bayala et al., 2014; Gong et al., 2015; Liu et al., 2016; Jiang et al., 2017; Fu et al., 2018; Chen et al., 2020a).

Gefitinib, a classic epidermal growth factor receptor (EGFR) tyrosine kinase receptor inhibitor, has been a bottleneck in the treatment of NSCLC due to the problem of drug resistance (Onitsuka et al., 2010; Sun et al., 2020). Studies have shown that METTL3 is highly expressed in NSCLC and promotes the occurrence and development of NSCLC by promoting tumor cell proliferation, EMT, invasion, metastasis, angiogenesis, and anti-apoptosis. Moreover, highly expressed METTL3 can increase autophagy in NSCLC by upregulating autophagy-related proteins ATG5 and ATG7, and eventually lead to resistance to gefitinib and other EGFR TKI drugs. β-elemene can directly inhibit the expression of METTL3, but not VIRMA and METTL14, causing the down-regulation of autophagy-related proteins LC3B, ATG5, and ATG7, thereby inhibiting autophagy, tumor cell proliferation and promoting cell apoptosis, and finally reversing the effect of gefitinib resistance in NSCLC (Liu et al., 2020). In addition, PTEN is a well-known tumor suppressor in various cancers and acts as a negative regulator of PI3K-Akt pathway. β-elemene can reduce the level of m6A modification of PTEN mRNA by downregulating the expression of METTL3, resulting in an increase in the expression of PTEN protein, which ultimately inhibits the growth of tumor cells and promotes apoptosis (Feng et al., 2022).

### Soy Isoflavones Genistein

Genistein is a major active factor in soy isoflavones, the most effective functional component in soy isoflavone products, and has a variety of physiological functions. Genistein is similar in structure to mammalian estrogen-estradiol, and has the diphenolic hydroxyl active group of estrogen. So genistein has various physiological activities such as estrogen-like activity.

Studies have shown that the antioxidant and anti-proliferative properties of soy isoflavones are the main reasons for their anti-cancer effects. Soy isoflavones have obvious therapeutic effects on breast cancer, colon cancer, lung cancer, prostate cancer, skin cancer, and leukemia. Soy isoflavones can also prevent the occurrence of ovarian cancer, colon cancer, stomach cancer, and prostate cancer (Suthar et al., 2001; Sarkar et al., 2006; Kaushik et al., 2018; Park et al., 2018; Imai-Sumida et al., 2020; Aboushanab et al., 2021). In addition, down-regulation of ALKBH5 expression enhanced the expression of mesenchymal phenotype markers α-smooth muscle actin and snail, which are factors that promote EMT. Genistein can increase the expression of ALKBH5, reduce the level of RNA m6A and inhibit EMT (Ning et al., 2020).

### Resveratrol

Resveratrol, a non-flavonoid polyphenolic organic compound, is a phytoalexin with the chemical formula C_14_H_12_O_3_. It can be synthesized in grape leaves and grape skins, and is a biologically active ingredient in wine and grape juice. Resveratrol has antioxidant, anti-inflammatory, anti-cancer and cardiovascular protection effects (Rauf et al., 2018a). The heavy metal cadmium (Cd) can promote the migration and invasion of colorectal cancer cells, and Cd could upregulate the expressions of N-cadherin, vimentin, and ZEB1 and downregulate the expression of E-cadherin in colorectal cancer cells. Resveratrol could reverse Cd-promoted migration, invasion, and EMT processes by modulating ZEB1 expression (Qian et al., 2021b). Moreover, resveratrol can reduce aflatoxin B1-induced ROS accumulation, and can also cause changes in m6A modification-related proteins, including: METTL3, FTO, YTHDF2 (Wu et al., 2020).

### Rhein

Extracted from the rhizome of Rheum palmatum, Rhein is the main bioactive component of Dahuang (rhubarb) therapeutic laxatives. In tumors, Rhein mainly inhibits cell proliferation, promotes cell apoptosis, inhibits tumor cell invasion, and metastasis, and enhances hypoxia tolerance, and so on. Its target signaling pathways include: PI3K-Akt, MAPK (ERKs, JNKs, P38), Wnt, NF-kB, et al. (Henamayee et al., 2020). Rhein was the first FTO-competitive inhibitor discovered, which can increase the level of m6A modification on intracellular target mRNA. In addition, Rhein can reversibly bind to FTO or ALKBH5 to form a complex and prevent recognition of intracellular m6A substrates (Chen et al., 2012; Zannella et al., 2021).

### Baicalin

Baicalin is a flavonoid compound extracted and isolated from the dry root of Scutellaria baicalensis Georgi (a dicotyledonous Lamiaceae plant), which has a variety of biological activities, including bacteriostatic, diuretic, anti-inflammatory, cholesterol-lowering, anti-thrombotic, anti-asthmatic, hemostatic, anti-allergic, and antispasmodic effects, and also has a strong anti-cancer biological effect (Wang et al., 2015; Gong et al., 2017; Lai et al., 2018).

Type 2 diabetes (T2D) is defined as a metabolic disorder characterized by hyperglycemia that can lead to abnormal organ metabolism or hormones, which in turn lead to a variety of diseases, including cancer. High glucose environment can induce hepatoma cells to up-regulate the expression of HKDC, which can promote the proliferation, invasion and metastasis of tumor cells by downregulating the HKDC/JAK2/STAT1/caspase-3 signaling pathway. Baicalin can reduce the m6A modification level of HKDC mRNA by inhibiting METTL3, which leads to the decrease of HKDC expression and the upregulation of HKDC/JAK2/STAT1/caspase-3 signaling pathway, and finally inhibits the invasion and metastasis of tumor cells (Jiang et al., 2022). In addition, Baicalein inhibits pancreatic cancer cell proliferation and invasion by inhibiting NEDD9 and its downstream Akt and ERK signaling pathways (Zhou et al., 2017).

### Others Natural Product

Humantenine, an indole alkaloid compound isolated from Gelsemium elegans, is a traditional medical herb with a drug use. Wu et al (2022) studies indicated that the humantenine leads to the colon cancer cell injury *via* affecting the gene transcriptome and m6A regulators, including RBM15, METTL3, YTHDF3, and ALKBH5 are up-regulated, and RBM15B, ZC3H13, hnRNPA2BA, YTHDC1, YTHDC2, YTHDF2, and IGF2BP3 are down-regulated.

Curcumin, a polyphenol extracted from turmeric in 1815, has attracted worldwide attention due to its biological activities (such as antioxidant, anti-inflammatory, antibacterial, and antiviral), among which its anticancer potential has been described the most, and the relevant anticancer mechanism is still being investigated (Mortezaee et al., 2019; Wang et al., 2021). Curcumin inhibits the occurrence and development of tumor cells by regulating the expression of growth factors, inflammatory factors, apoptosis-related proteins, protein phosphokinases, and receptors, and cell proliferation-related genes, such as FGFs, VEGF, EGF/EGFR, TNF, IFN, ILs, Caspase-3/6/8/10, FADD, MAPK, JNK, IKK, Survivin, MCL-1, BCL-xL, cIAP-1/2, BCL-2, c-Myc, PCNA, Cyclin D1, etc (Giordano and Tommonaro, 2019). Unfortunately, there is still no relevant report on the relationship between curcumin and m6A enzyme, which also provides a broad space for us to explore the correlation between curcumin and m6A enzyme and its anti-cancer mechanism.

Chrysin (5,7-dihydroxyflavonoid) is a natural, bioactive dietary flavonoid commonly found in a variety of plant extracts, including chamomile, pleurotusostreatus, and honeycombs, as well as in honey and propolis, which has a significant medicinal function and economic value. Chrysin has a variety of biological properties, including anti-cancer, antioxidant, anti-inflammatory, antibacterial, and other effects. Several recent studies have reported that chrysin exerts its anti-cancer effects on endometrial cancer, gastric cancer, breast, lung, cervical, bladder, breast cancer, and colorectal cancer, etc. *via* selectively inhibiting various cell signaling pathways, such as: Akt/mTOR, JNK1/2, ERK1/2, NF-κB, etc., and promoting tumor cells apoptosis and autophagy (Xia et al., 2015a; Xia et al., 2015b; Roy et al., 2019; He et al., 2021). However, the regulatory relationship between chrysin and m6A enzyme has not been reported.

**FIGURE 2:**
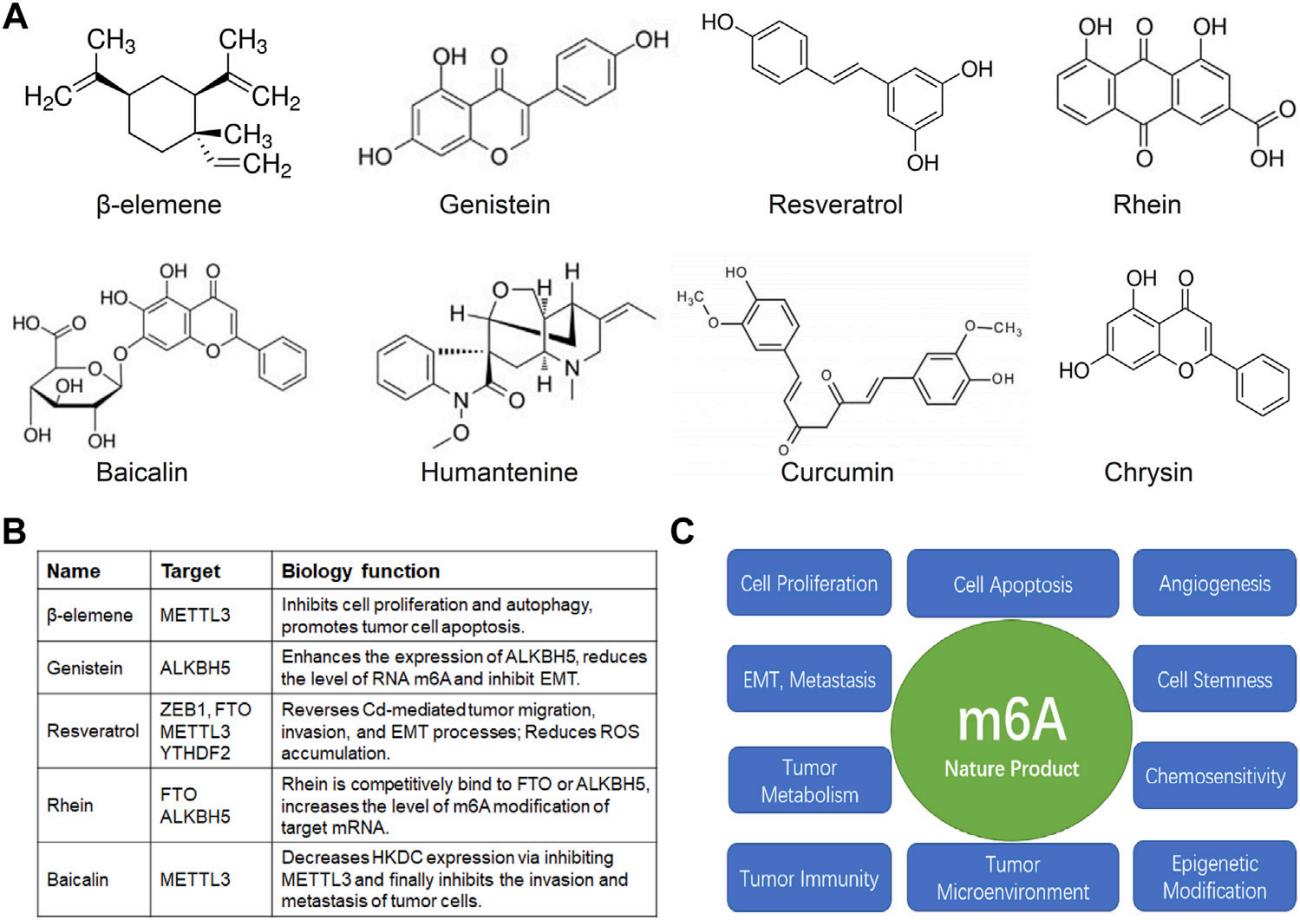
Natural products and m6A in anti-cancer research. **(A)** The structure of the common natural anticancer products, follow as: β-elemene, genistein, resveratrol, rhein, baicalin, humantenine, curcumin, chrysin. **(B)** The anti-cancer effects of β-elemene, genistein, resveratrol, rhein, baicalin depended on m6A enzyme. **(C)** The possible mechanism of nature products mediated m6A modification in tumor cell proliferation, apoptosis, angiogenesis, EMT, invasion, metastasis, maintenance of tumor cell stemness, tumor metabolism, chemosensitivity, tumor immunity, tumor microenvironment, and epigenetic modification.

In addition, there are a number of natural products with anticancer activities, such as: cucurbitacin B, ailanthone, fucoidan, casearlucin A, Wan-Nian-Qing prescription, bruceine D, cinnamaldehyde, fisetin, quercetin, betulinic acid, astragalus polysaccharide, ginsenosides, panax notoginseng saponins, and so on (Wong et al., 2015; Syed et al., 2016; Rauf et al., 2018b; Li et al., 2020c; Jiang et al., 2021a; Huang et al., 2021c; Imran et al., 2021; Yao et al., 2021). By regulating the whole process of tumor occurrence and development, such as cell cycle, apoptosis, autophagy, invasion, and migration, and cell metabolic reprogramming, etc. (Figure 2), these natural products have broad research value and clinical treatment in the field of anti-cancer.

## Conclusion

More and more studies have shown that m6A modification plays a key role in the tumorigenesis and progression of various cancers, and the abnormal expression of m6A modification-related proteins is closely related to the occurrence and development of tumors. Natural products have broad prospects and applications in the field of anti-tumor research. At present, some natural products have played an important role in clinical applications. There are relatively few studies on the regulation of m6A modification by natural products, and need more extensive and in-depth research in the field of anti-tumor. The role of natural products in tumors needs further study, m6A modification in cell proliferation, apoptosis, angiogenesis, EMT, invasion, metastasis, maintenance of tumor cell stemness, tumor metabolism, chemosensitivity, tumor immunity, tumor microenvironment, and the roles and mechanisms of epigenetic modification still need to be further explored.
